# Meeting Report: Investigating Indoor Air

**Published:** 2005-03

**Authors:** Ernie Hood

Americans spend about 85–95% of their time indoors, and in recent years, the indoor environment and its potential effects on health have become the subject of increasing research attention. Seeking to explore the existing knowledge and stimulate new ideas for action, the nation’s top public health officer convened a gathering of more than 300 experts from government, academia, the building materials and design industries, and public interest groups for a two-day conference in January 2005.

The Surgeon General’s Workshop on Healthy Indoor Environment began with presentations of the scientific evidence that exposure to polluted indoor air is making many people sick. Those exposures include chemicals (such as volatile organic compounds and pesticides), biological agents, other particulates, environmental tobacco smoke, excessive dampness, and poor ergonomic, noise, thermal, and lighting conditions. Eileen Storey, director of the University of Connecticut Center for Indoor Environments, said surveys indicate that on average 40–55% of office occupants experience some degree of so-called sick building symptoms (such as headache, cough, wheezing, and fatigue) on a weekly basis.

Speakers discussed the 2004 Institute of Medicine report *Damp Indoor Spaces and Health*, an exhaustive literature review which concluded that excessive indoor dampness and fungal growth are consistently and convincingly associated with respiratory health effects, including asthma. The report delineated research needs in the area, including reproducible, validated measurement and risk assessment methods. Peyton Eggleston, a professor of pediatrics and immunology at the Johns Hopkins School of Medicine, said, “We [also] need an intervention study that will take a damp building, remediate it, and show in a rigorous scientific way that there is both a measurable environmental impact and a measurable health impact.”

There was also a consistent call to implement good practices based on current knowledge, and to express the benefits of improving indoor air quality in terms of economic value, so building owners, operators, and occupants can appreciate that such investments make bottom-line sense. Storey presented estimates from the September 2002 *American Journal of Public Health* that improving indoor air may save businesses $5–75 billion annually through fewer sick building symptoms, communicable respiratory diseases, allergies, and asthma attacks, with concomitant gains in productivity. “If we calculate it in those ways,” said Storey, “people will immediately say, ‘It’s worth it to run my building in a way that people are not going to get sick.’” Several participants cited the need for well-defined standards of what constitutes a healthy indoor environment, perhaps including a building rating system. Several government agencies represented at the workshop currently have research initiatives involving the indoor environment. Surgeon general Richard Carmona urged more collaboration, not only among federal agencies, but also in research and intervention partnerships with academia and the building professions. “The issue of a healthy indoor environment is key to improving the health of the American people,” he said. “Clearly the time has come for action in this area.”

## Figures and Tables

**Figure:**
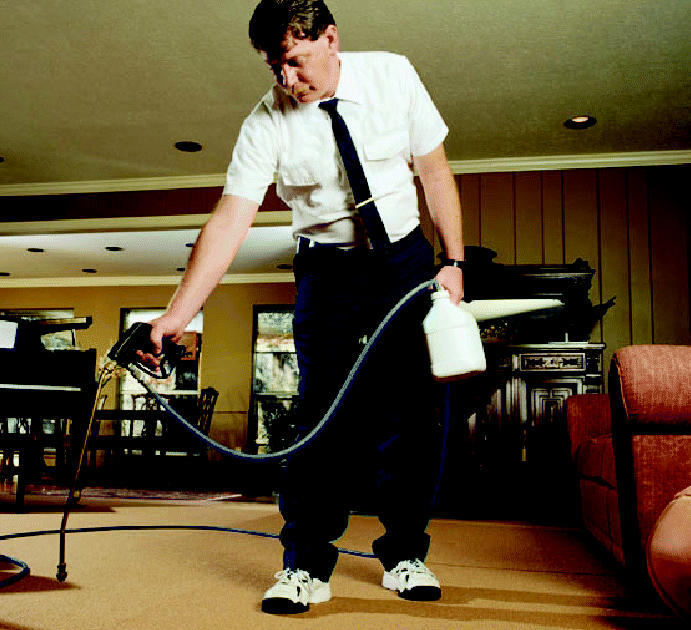
**Focus on fumes.** A recent workshop reviewed the many sources of indoor air pollution and what can be done about them.

